# Supplementary material for: Oxygen Vacancies on Hydrated Anatase (101) Surfaces: Insights from Classical and Ab Initio Molecular Dynamics Simulations
Source: Nanomaterials (Basel). 2025 Feb 27;15(5):364. doi: 10.3390/nano15050364 (PMC11901595; doi:10.3390/nano15050364)
Supplement: Supplementary file 1 [file nanomaterials-15-00364-s001.zip › nanomaterials-3463160-supplementary.pdf]

Supporting information for publication: Oxygen vacancies on hydrated anatase (101) surfaces: Insights from classical and ab initio molecular dynamics simulations

Fredrik Grote and Alexander P. Lyubartsev*

Department of Chemistry, Stockholm University,

SE 106 91, Stockholm, Sweden

tel. +46-8161193

E-mail: alexander.lyubartsev@mmk.su.se

Details on basis set and convergence of plane wave cutoff

The GPW method uses atom centered gaussian basis functions to expand Kohn-Sham orbitals and an auxillary plane wave basis set for the electron density. In this work we used the MOLOPT-DZVP gaussian basis set developed by VandeVondele and Hutter.[1] In order to monitor convergence of the total energy and distribution of gaussian functions onto the four different levels of the multigrid we performed a number of test runs varying the plane wave cutoff and relative cutoff (parameters CUTOFF and REL_CUTOFF in CP2K input file). Figure S1 shows the total energy and gaussian count as function of the cutoff and relative cutoff. These results motivates using a 300 Ry plane wave cutoff and 30 Ry relative cutoff providing converged total energy as well as even distribution of gaussians on the different levels of the multigrid.

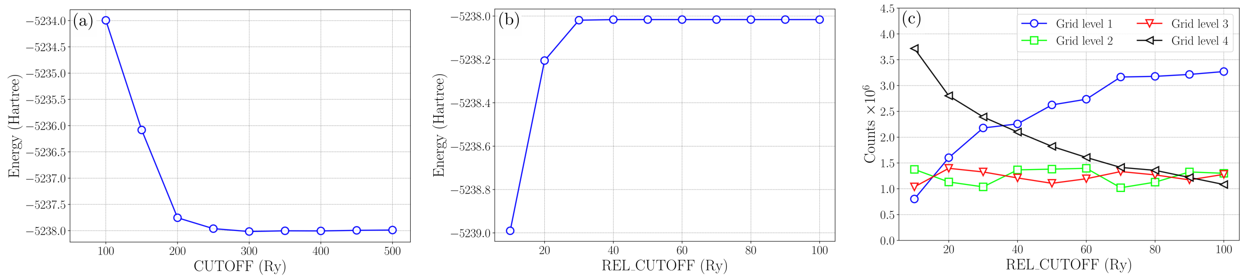

Figure S1: Convergence of total energy with respect to (a) plane wave cutoff, (b) relative cutoff and (c) distribution of gaussians on different levels of the multigrid.

References

(1) VandeVondele, J.; Hutter, J. Gaussian basis sets for accurate calculations on molecular systems in gas and condensed phases. *J. Chem. Phys.* **2007**, *127*.
